# X-ray computed tomography (XCT) and chemical analysis (EDX and XRF) used in conjunction for cultural conservation: the case of the earliest scientifically described dinosaur *Megalosaurus bucklandii*

**DOI:** 10.1186/s40494-018-0223-0

**Published:** 2018-10-03

**Authors:** P. F. Wilson, M. P. Smith, J. Hay, J. M. Warnett, A. Attridge, M. A. Williams

**Affiliations:** 1 Warwick Manufacturing Group (WMG)-University of Warwick, Coventry, UK; 2 Oxford University Museum of Natural History (OUMNH)-University of Oxford, Oxford, UK

**Keywords:** *Megalosaurus*, Conservation, X-ray computed tomography, Dinosaur, Heritage, 3D printing, XRF, EDX

## Abstract

This paper demonstrates the combined use of X-ray computed tomography (XCT), energy dispersive X-ray spectroscopy (EDX) and X-ray fluorescence (XRF) to evaluate the conservational history of the dentary (lower jaw) of *Megalosaurus bucklandii* Mantell, 1827, the first scientifically described dinosaur. Previous analysis using XCT revealed that the specimen had undergone at least two phases of repair using two different kinds of plaster, although their composition remained undetermined. Additional chemical analysis using EDX and XRF has allowed the determination of the composition of these unidentified plasters, revealing that they are of similar composition, composed dominantly of ‘plaster of Paris’ mixed with quartz sand and calcite, potentially from the matrix material of the Stonesfield Slate, with the trace presence of chlorine. One of the plasters unusually contains the pigment minium (naturally occurring lead tetroxide; Pb_2_^2+^Pb^4+^O_4_) whilst the other seems to have an additional coating of barium hydroxide (Ba(OH)_2_), indicating that these likely represent two separate stages of repair. The potential of this combined approach for evaluating problematic museum objects for conservation is further discussed as is its usage in cultural heritage today.

## Introduction

The need to preserve and care for collections is of key importance to any museum, so that objects of cultural and natural significance can be safeguarded for future generations to appreciate and, most importantly, to learn from [1]. This goal is a fundamental underlying principle of museums as institutions. As a result, museums are required to care for their collections in order to ensure their legacy for future generations, a practice as old as the museum itself [2, 3]. This task is mandatory for ensuring that historical objects of interest to science, history and art remain unblemished by poor storage conditions, incidental chemical degradation or general wear and tear over time through handling, display and storage [3–5]. Over the years, best practices for effective treatment of museum artefacts have been collectively developed by a variety of museum institutions under the umbrella-term conservation, defined by the International Council of Museums Conservation Council (ICOM-CC) as:

*“All measures and actions aimed at safeguarding tangible cultural heritage while ensuring its accessibility to present and future generations.”* [6]

Through conservation, an institution can protect its valuable artefacts from the effects of time and without an effective conservation strategy, the core purpose of the museum, to protect and conserve cultural and natural heritage, is rendered null and void [7]. Conservation can be broadly broken down into three different categories: preventative conservation, whereby artefacts are kept safe through control of their environment rather than direct intervention; remedial conservation, where artefacts are treated to stabilise them and prevent further damage and degradation in the immediate future; and restoration, where the artefact is restored to prime condition [3, 6, 7]. Given the sensitivity of different materials to fluctuations in temperature, humidity and light and how they age [7, 8], it is the duty of conservators to know the best conservational methods to use, particularly when carrying out remedial conservation or restoration to ensure that the object remains chemically and physically stable for many years to come, and what treatments are reversible [3]. For this reason, keeping records of the treatment that a specific artefact has undergone, the materials used and when work was carried out is extremely important to ensuring that future treatments do not cause harm by utilising incompatible materials that may result in incidental, unintentional damage to artefacts in the long term, as is the case with the well-known Bernissart dinosaur collection at the Royal Belgian Institute of Natural Sciences in Belgium, discovered in 1877. This collection, in order to strengthen the already fragile bones, was treated with varnishes and glues to consolidate the specimen and now provide continuous conservational concerns some 150 years later [9].

Conservation treatment records do not, however, always survive through the years and may be lost, damaged or even destroyed through disasters such as fire or bombing, an issue particularly prevalent in institutions with a pre-WWII history, such as Bristol Museum and Art Gallery [10–12]. They may also simply fail to be noted at the time of conservation, an issue prevalent in older conservational practice where craft repairers responsible for restoration rarely kept records of what treatments were applied [3, 13, 14]. This highlights a significant problem, that without a solid knowledge of what techniques and materials have been used to ensure an objects’s survival, conservators are left unsure of the extent of repair that a specimen has undergone and the materials used, making it much more difficult to assess the best methods to be used in order to stabilise or restore an object. Another potential issue is that of fakes, forgeries and genuine-but-repaired objects, which on occasion can trick even the most astute subject experts, causing potential embarrassment to institutions and leading to loss of valuable funding through fraud [15, 16]. Thus, a non-invasive, non-destructive, repeatable method of uncovering the conservational history of an object or artefact is needed in order to ascertain what treatment an object has undergone within its residence time at a museum, or before it arrived.

**Fig. 1:**
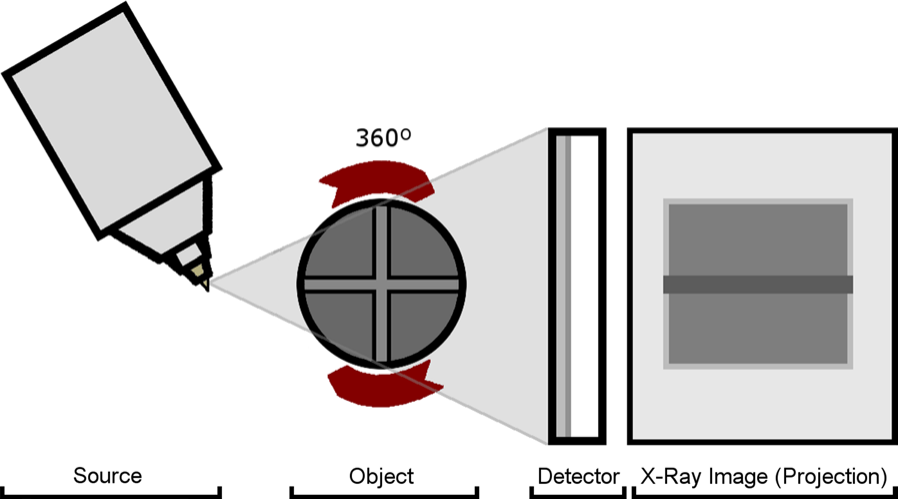
The arrangement of components within an X-ray computed tomography (XCT) scanner. The scanning system is composed of an X-ray source, an object on a rotating platform and a flat detector. These systems are used to collect X-ray radiographs from 360º around an object

Many authors have discussed the use of X-ray computed tomography (XCT) for conservation purposes, giving museums and cultural institutions the ability to explore the sub-surface construction of their precious objects and ascertain aspects of their conservation [11, 17, 18]. XCT is an approach that utilises X-rays in a similar manner to conventional X-ray radiography, generating a set of radiographs of the object which reveal hidden, internal structures [19, 20]. This is achieved using an XCT scanner (Fig. 1), in which the object is placed between an X-ray source and a detector, after which a beam of x-rays, either fan or cone shaped, is fired through the object and the amount of the x-rays attenuated by the object, a value that is related to absorptive properties of the material with regard to x-rays, recorded by the detector plate [11, 20–22]. The object is then rotated by a small increment and the process is repeated, until X-ray projections of the object from all angles through 360º are collected [23]. These are then converted into a three-dimensional volume through a process known as reconstruction, which combines the projections together to create a greyscale volume consisting of voxels, these greyscale values being representative of the relative attenuation of the x-rays, a product of a number of properties of the material, such as its density, chemical composition and thickness. Brighter grey values indicate highly attenuating material and darker grey values represent less attenuating material [20, 21].

This approach is growing in accessibility and popularity, and has been used for a wide variety of conservation approaches, including testing methods of restoring stonework and evaluating methods of its conservation [24, 25], evaluating the subsurface structure of artworks [26, 27], identifying the internal consistency and subsurface make-up of heritage artefacts [11, 15, 17, 28], as a pre-stage to sample preparation [11, 29] and even in identifying forgeries and repaired artefacts that would have otherwise evaded detection [15, 30]. The major benefit that this approach provides is the ability to image the internal structure of an object non-destructively and with extremely low-risk to the artefact itself. It also provides the ability to work at a wide variety of scales, ranging from medical XCT imaging for larger objects down to synchrotron XCT for objects at the sub-millimetric scale [22]. As a result, XCT imaging is a technique with significant potential in the non-destructive analysis of the internal structure and make-up of museum objects [11]. However, one limitation is that while it is capable of highlighting secondary structures and characterising areas of prior conservation, the technique is less adept at identifying the composition of the different materials within the object, although dual-energy CT is an approach can be utilised to gain a better understanding of the this [31, 32]. This approach utilises two separate X-ray tubes simultaneously at different voltage values (kV) to identify relative compositional differences, as some materials attenuate differently at different scanning energies and is commonly used in medicine [33, 34] and occasionally in cultural heritage [32, 35, 36]. However, this does require some prior knowledge and categorisation of how these materials behave at different kV values, although this has been categorised for a number of different materials [33, 37]. Unfortunately, this makes this useful non-destructive approach of limited use in dealing with unknown or uncertain materials. Given that the composition of these materials is a key aspect of conservation that strongly influences the types of treatments that can be safely applied to objects at risk. Thus XCT should be combined with other approaches that directly complement its relative strengths and weaknesses.

Here we demonstrate an example of how XCT can be combined with chemical analysis approaches, such as energy dispersive X-ray spectroscopy (EDX) and X-ray fluorescence (XRF), to better understand the composition of repair plasters previously found in the lectotype dentary (lower jaw) of the first scientifically described dinosaur, *Megalosaurus bucklandii* Mantell, 1827 [38], a unique historical specimen housed at Oxford University Museum of Natural History (OUMNH) [39, 40]. The 167 million year old fossil, which was discovered in a limestone quarry over 200 years ago is known to have undergone extensive repair throughout its long history in the collections of Buckland himself, Christ Church College of Anatomy and the modern OUMNH, with significant amounts of plaster being used to repair broken portions. A previous study using XCT [18] elucidated that the specimen had undergone significant treatment with plaster, previously of unknown extent, in two separate phases. Both of these had different physical characteristics, one being slightly less dense and the other containing fine, highly dense particles evenly disseminated throughout. What these plasters are made of was unknown and there were few records of what repair materials were kept historically in the museum. As a result, this iconic specimen provides an excellent case study into how XCT can be combined with chemical analysis techniques to provide a powerful approach for analysing the prior conservation of problematic items from the past.

## Methods and materials

### Aim of study

The aim of the study is to examine the conservational history of the *M. bucklandii* lectotype dentary in order to ascertain what treatments it has undergone in the past and to attempt to work out the relative timing of these. The study utilises a number of methods used to determine the chemistry of the repair material used in the object, including Energy-Dispersive X-ray spectroscopy (EDX) and X-ray fluorescence (XRF).

### Materials

**Fig. 2:**
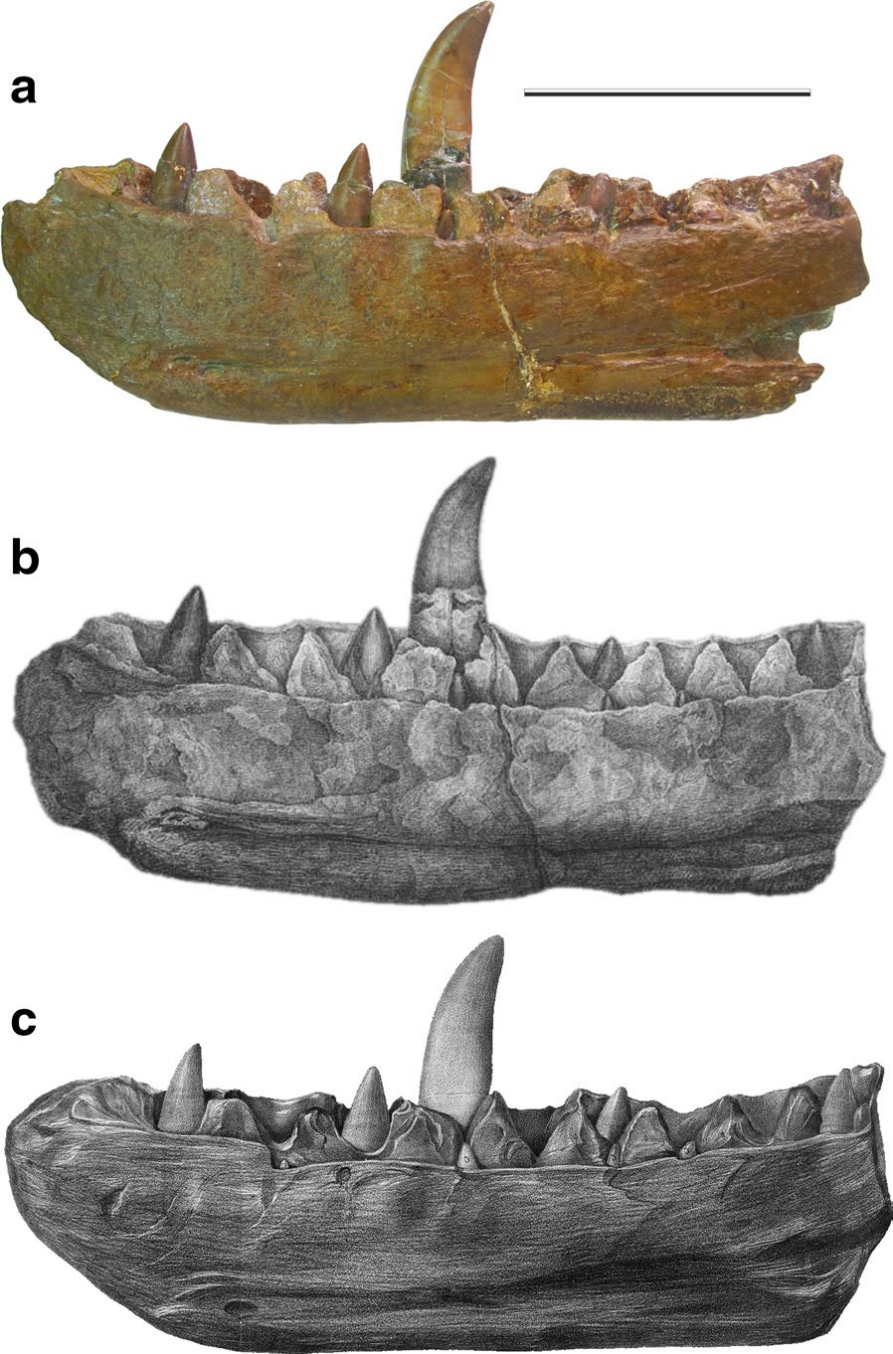
Illustrations of the lectotype dentary specimen of *Megalosaurus bucklandii* Mantell, 1827, from 1824 to the present day. **a** Modern photograph of the lectotype dentary. Scale bar equals 10 cm. **b** Lithograph from Buckland [39] and Owen [43], the first appearance of the dentary in literature and museum records. (Plate XLI, Fig. 1), which does not appear to accurately represent the actual specimen. **c** Appearance in Owen [44], more accurately capturing the shape of the specimen with some tooth damage missing in later illustrations (Plate 33, Fig. 1)

All of the materials used in this project are derived from the right dentary of *M. bucklandii*, the lectotype specimen of the species, housed at Oxford University Museum of Natural History (OUMNH J.13505) (Fig. 2a). The dentary is associated with two thin slabs of limestone (J.13505b and J.13505c), which are parts of the same specimen, and both of which have an impression of the dentary together with a small amount of bone material still attached from the lateral and medial surfaces of the specimen. The specimen is preserved as primary calcium phosphate with infill of any decayed organic matter by secondary, diagenetic minerals.

The specimen itself is from the Middle Jurassic ‘Stonesfield Slate’ of the Taynton Limestone Formation, and was found in a quarry in Stonesfield, Oxfordshire on an unknown date in the late 18th century. It has a long and, to some extent, uncertain, history [41, 42]. The dentary is first noted in the collection at the Christ Church Anatomy School, Oxford, in 1797, arriving a few years before William Buckland, the man who was to first describe the specimen [42]. It was not, however, until 1824 that Buckland first formally described the fossil bones of *Megalosaurus* [39], which was later given the specific name *bucklandii* by Gideon Mantell [38]. The position of *M. bucklandii* in history as the world’s first scientifically described dinosaur was cemented by Richard Owen in the formal taxonomic description of the Dinosauria alongside *Iguanodon* and *Hylaeosaurus* [43]. The early history of *M. bucklandii* type material is more fully documented by [42].

### Methods

#### Energy dispersive X-ray spectroscopy (EDX) and X-ray fluorescence (XRF)

In order to determine the composition of the materials used in the *M. bucklandii* dentary, samples of plaster from across the specimen were taken and subjected to EDX analysis. A total of seven samples of plaster material from across the specimen based upon their known locations from the CT data were taken, five from one of the suspected plaster materials (M1) and the two from the other (M2). A smaller number of samples were taken from this second material on the advice of the museum conservator, who did not wish to carry out any further destructive sampling on this very visible part of the specimen. These were then affixed to an SEM stub via carbon tape and gold-coated to a thickness of 5 nm. The samples were placed in a Zeiss Sigma SEM at AAMC (WMG-University of Warwick) and their composition analysed using EDX mapping.

A further, larger sample was taken from the plaster of the ventro-posterior portion of the dentary (M1) to explore the bulk composition of the plaster material. This sample was cut and mounted on a glass microscope slide in order for its composition to be examined via light microscopy and X-ray fluorescence spectroscopy (XRF). XRF imaging was done using a Bruker M4 Tornado at AAMC and the slide was examined under plane-polarized and cross-polarized light using a standard petrological microscope to confirm the results of the analysis.

## Prior knowledge of repair

**Fig. 3:**
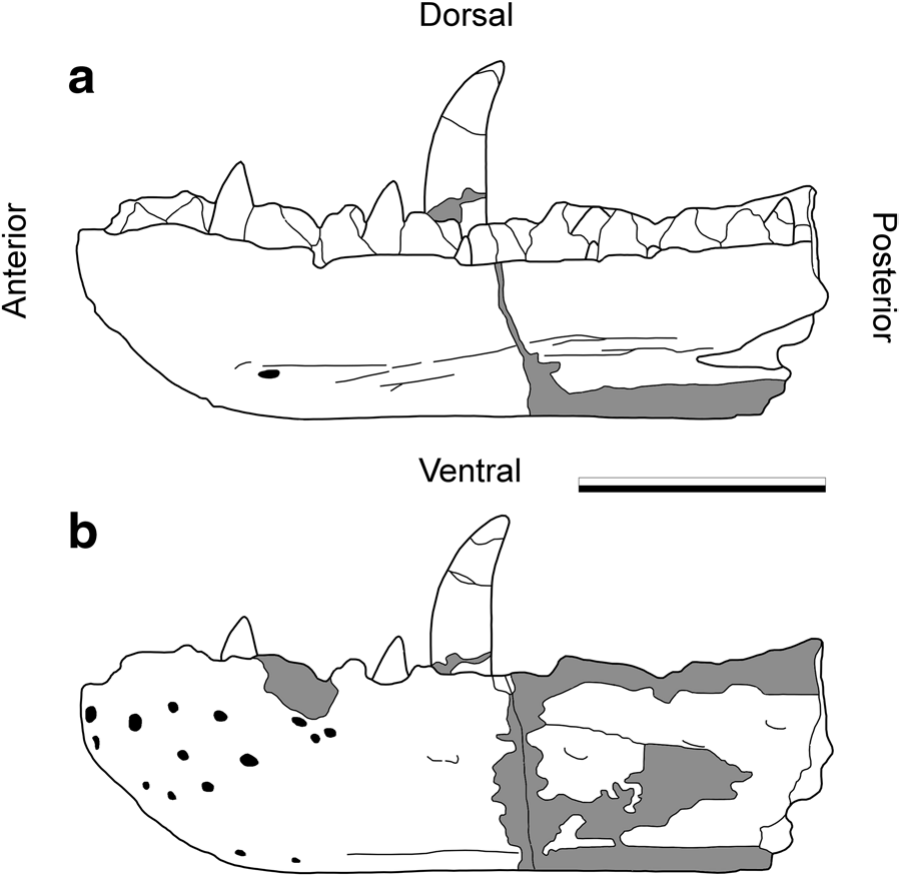
The location of suspected plaster repair identified by Benson et al. [41] on the *Megalosaurus bucklandii* lectotype dentary. Grey areas indicate those suspected to be composed of plaster. Modified from [41] **a** medial surface and **b** lateral surface. Scale bar is 10 cm

In spite of the scientific and cultural significance of the lectotype dentary of *M. bucklandii*, very little is known about its conservation history. Since its first appearance in the collections in 1797 [42], few records have been found that indicate the level of repair that the specimen has undergone in the 220 or so years it has been in the collections at the University of Oxford. Its first properly figured appearances in Buckland [39] in 1824 and Owen [43] in 1842 lack any indication of repair, [41]. The specimen here is complete, the only noticeable damage being depicted in [39] where there is a large dorso-ventral crack located just behind the prominent, mature tooth. However in this figure, this crack is only clearly depicted on the medial surface of the jaw (Plate XLI, Fig. 2b) and is absent from the lateral surface suggesting that it was only superficial rather than the clear break it is today (Fig. 3). Also notable is the strange shape of the specimen compared to its modern appearance, either indicative of damage over time or a fact that this early lithograph does not accurately depict the fossil.

The latter theory is supported by J. Erxleben’s depiction of the specimen in Owen’s *A history of British fossil reptiles* [44] (Plate 33, Fig. 2c) which more accurately resembles its current state. The aforementioned crack in the earlier depiction is not seen in this figure so cannot be corroborated, but a horizontal fracture on the large, prominent tooth is present, and provides a maximum age for its origin (Fig. 3). The specimen is next figured in an article by Phillips [45] in 1871, though the complete specimen is depicted as part of a diagnostic restoration of the skull, rather than as a faithful lithograph (Diagram LVII). A single diagram of the largest mature tooth is featured (Diagram LVI) which shows no cracks, contradicting the figure found in Owen [44]. It is likely that the damage may have been omitted from the Phillips illustration [45] to focus on the demonstration of its anatomy. Additionally, limited museum records seem to indicate that some form of repair was undertaken between 1927 and 1931 when the specimen was first put on display, although the treatments and materials used and the extent of repair remained undocumented. As a result, little is known about the provenance and composition of the materials used to repair the *M. bucklandii* specimen.

Previously documented in [18, 41] is the common knowledge that the specimen has undergone a significant amount of restoration using plaster to infill broken parts of the jaw. This is best documented by Benson et al. [41] who provided a diagram (Fig. 3) of the places suspected to be plaster. From this diagram it can be observed that the majority of the plaster repair undertaken is on the posterior portion of the specimen, particularly infilling the fracture between the anterior and posterior part of the jaw, which were presumably broken into separate pieces at some point in the specimen’s history (Fig. 3a, b). This plaster also replaces a large portion of the ventral surface posterior of this fracture, material that was presumably too fragmented to be used in repair. A large amount of repair has also been undertaken on the lateral surface, particularly along the dorsal margin posterior to the central fracture and on the lateral surface, extending from the central fracture to replace more damaged surface material. Another small area of plaster can be found on the anterior part of the lateral surface, replacing part of the tooth row on the dorsal surface. The fifth tooth, the largest and most prominent, has been glued back together multiple times during the specimen’s history, most recently using a conservation-grade acrylic resin (Paraloid B72).

## Previous understanding of plaster conservation using X-ray computed tomography (XCT)

**Fig. 4:**
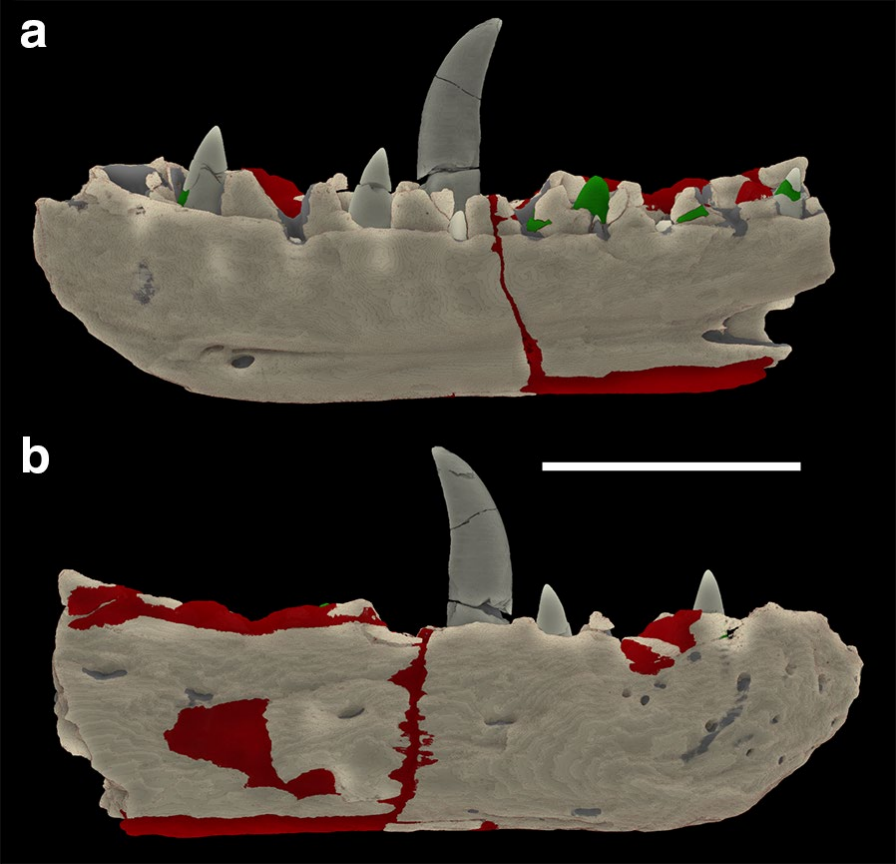
Identification of repair using X-ray computed tomography (XCT) from the *Megalosaurus bucklandii* type specimen [18]. **a** Medial surface of jaw and **b** lateral surface of jaw. Areas marked red indicate plaster of Material 1 (M1) and areas marked green indicate plaster of Material 2 (M2). Images rendered in Drishti. Scale bar is 10 cm. Modified from [18]

**Fig. 5:**
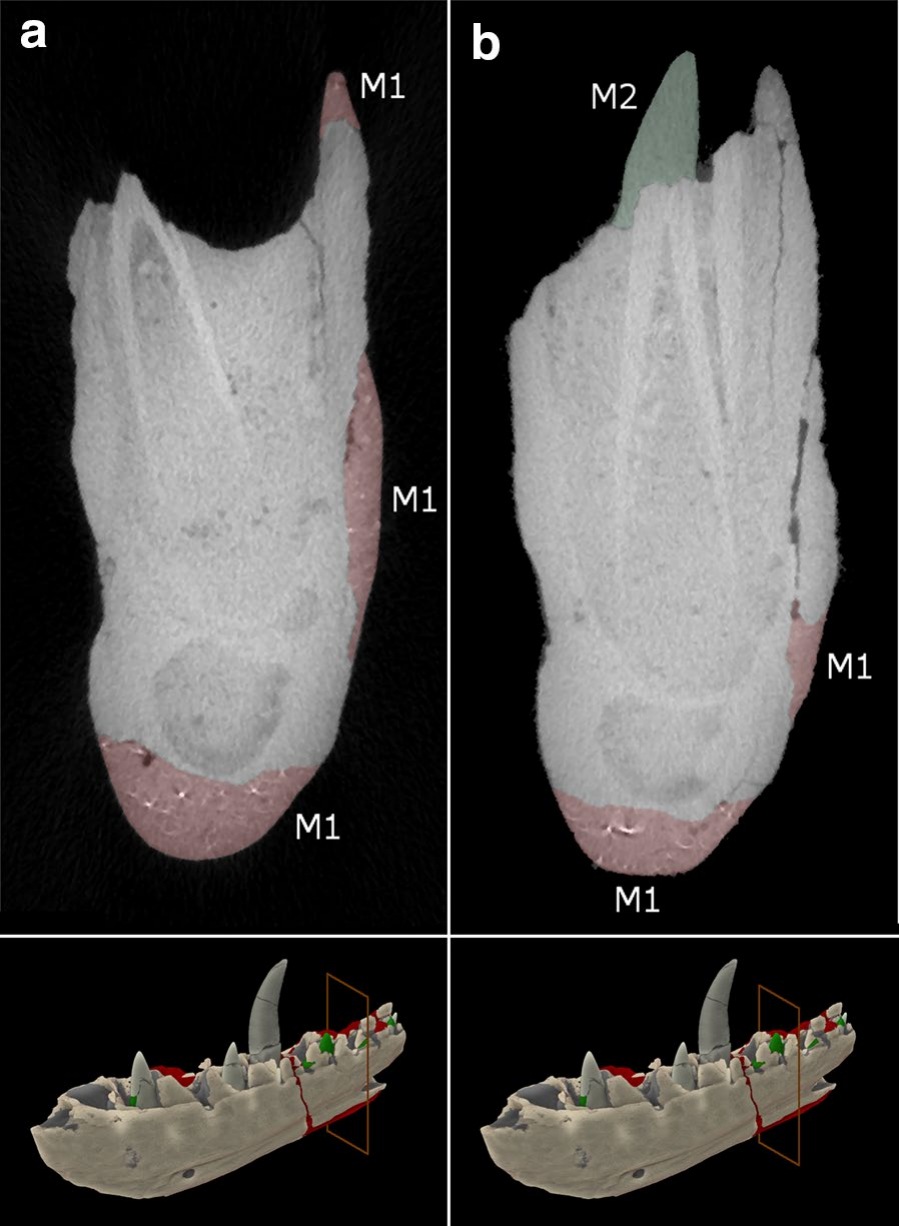
The internal structure of the *Megalosaurus bucklandii* lectotype dentary [18]. **a** Slice showing the depth of plaster of Material 1 (M1) and slice location. **b** Slice showing the positions of both Material 1 (M1), Material 2 (M2) and slice location
- Material 2 (M2-green): M2 makes up approximately 0.3% of the total volume of the jaw and by contrast is more homogeneous, lacking the highly attenuating particles while exhibiting darker grey values than M1 (Fig. 5b). This material can only be found along the tooth row where it repairs small pieces of damage to the alveoli and on the teeth, where it replaces broken crowns, supports severed crowns and in one case, replaces a completely missing, slightly offset crown (Fig. 5b).

The overall nature of the nature and distribution of the plaster repair in *M. bucklandii*, is covered in detail by [18] and the results from that study are outlined in brief here. Overall, the plaster replacement in *M. bucklandii* is fairly substantial (Fig. 4). Two plasters of notably different appearance can be clearly observed from the CT data:

- Material 1 (M1-red): M1 makes up approximately 3.5% of the total volume of the specimen and can be readily distinguished from the second material by its slightly brighter grey values and the presence of small (< 1 mm) highly attenuating particles, evenly distributed throughout the plaster (Fig. 5a). This material makes up the bulk of plaster repair carried out, being used on the medial and lateral sides, along the ventro-posterior portion of the specimen and on some parts of the tooth row.

Overall, the amount of plaster conservation is thought to be less than previously expected [41], revealed with great clarity thanks to the application of XCT technology.

## Inspection using energy-dispersive X-ray spectroscopy (edx) and X-ray fluorescence (xrf)

### EDX inspection

**Fig. 6:**
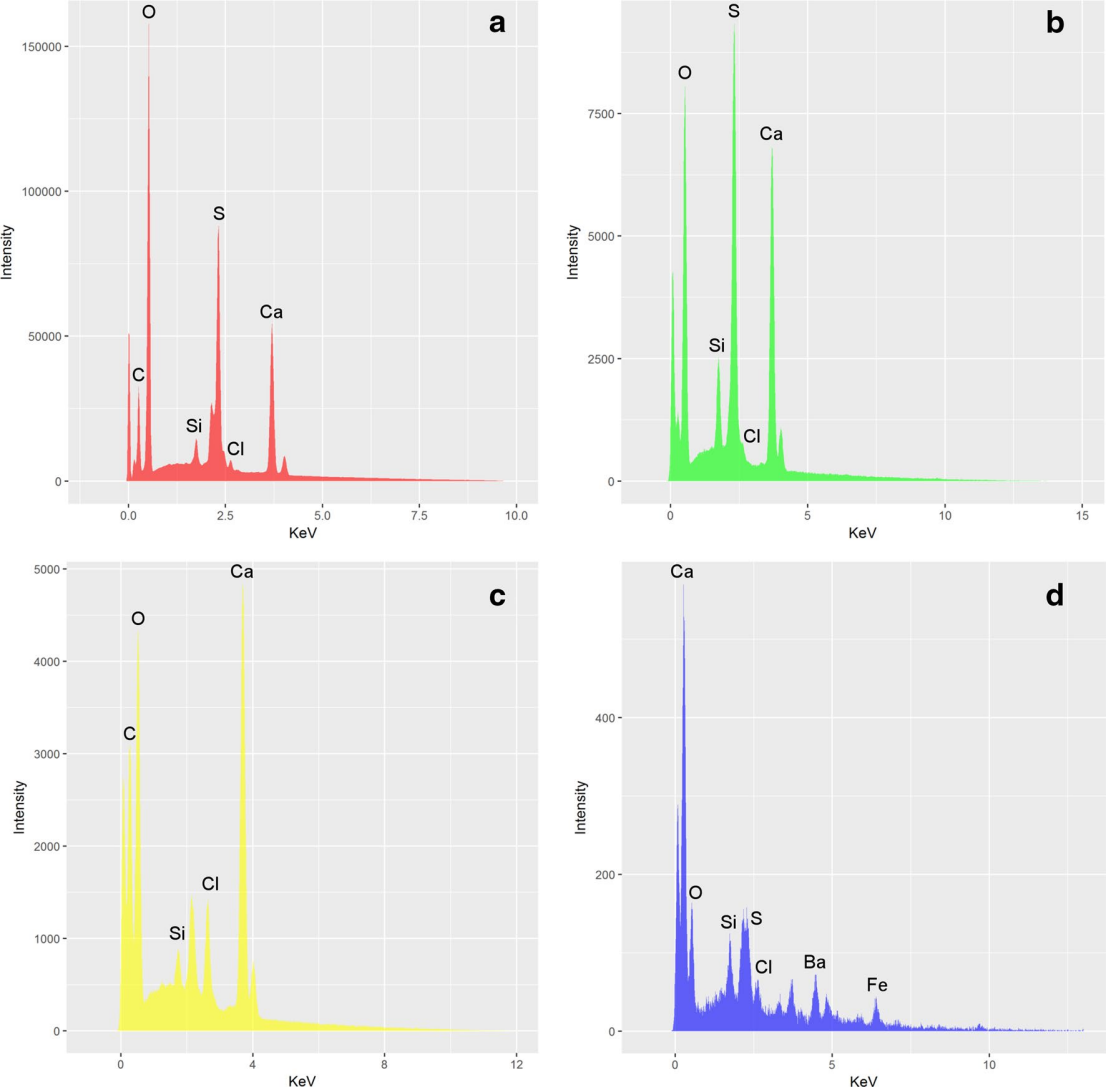
EDX plots of the two plaster materials. **a** Typical composition of Material 1 (M1), **b** typical composition of Material 2 (M2), **c** plaster containing large amounts of chlorine (Cl) (M2), **d** plaster sample containing elements such as barium (Ba) and iron (Fe) (M2). Gold (Au) peaks are unmarked, being associated with the standard approach of applying a gold coating to minimise charging

Overall, the composition of the plaster of both M1 and M2 replacements appear to be fairly similar (Fig. 6). The elemental spectra of the sampled plaster shows that the plaster is dominated, as expected, by oxygen (O), calcium (Ca) and sulphur (S), which make up the majority of the composition of the sampled plasters (Fig. 6a, b). This is consistent with the repair material being gypsum-based (CaSO_4_·2H_2_O), more commonly known as ‘Plaster of Paris’. The composition of the plaster is impure, with many additional elements that may not be accounted for in a composition of pure Plaster of Paris. The presence of silicon (Si) is chief among these and can be found in many of the plaster samples and is likely indicative of silica sand (SiO_2_). Exposed areas of plaster beneath the surface confirm this interpretation, with extremely fine grains (< 0.5 mm) of reddish brown sand being distributed throughout the material. Another unusual element that is present is carbon (C). This can most likely be attributed to a coating on the plaster, confirmed to be nearly pure carbon and is likely to be representative of an organic compound coating, such as shellac. However given that carbon tape was used in mounting the samples, this could also be another explanatory source, but considering that C was not present in every sample, this seems unlikely. The presence of this compound is unusual in that the majority of samples taken were examined on the freshly exposed surface of the fragments, but could likely be attributed to the coating agent sinking into the underlying plaster when it was applied, a commonly documented occurrence in plaster conservation (C. Hubbard and V. Borges, pers. comm.). Trace amounts of chlorine are present in a majority of samples (Fig. 6c) and could represent some form of contamination over the specimen’s life time. However, the origin of this chlorine is uncertain and could potentially be derived from cleaning agents applied to the specimen, from handling or atmospheric contamination. Similarly, small concentrations of iron (Fe) are found within a few samples and could represent isolated grains of elemental iron and/or oxides of iron mixed in with plaster, likely derived from the sand.

This composition is broadly similar in both M1 and M2, with a few minor differences. One sample contained significant quantities of barium (Ba) (Fig. 6d). The presence of barium is very unusual for plaster, but could represent barium hydroxide (Ba(OH)_2_) given the presence of oxygen (hydrogen (H) cannot be detected using SEM or EDX equipment). Barium hydroxide has historically been used as a coating for plaster as a consolidant and sealant to ensure that it remains impermeable and this is a likely origin for this element (C. Hubbard and V. Borges, pers. comm.). Given that this particular sample was taken from just below the plasters surface, as with the shellac coating noted above, it has likely leached into the plaster below. This is only found in one sample however and could represent a specific application to a certain part of plaster to strengthen it. This represents the only major compositional difference between the two plasters.

### XRF inspection

**Fig. 7:**
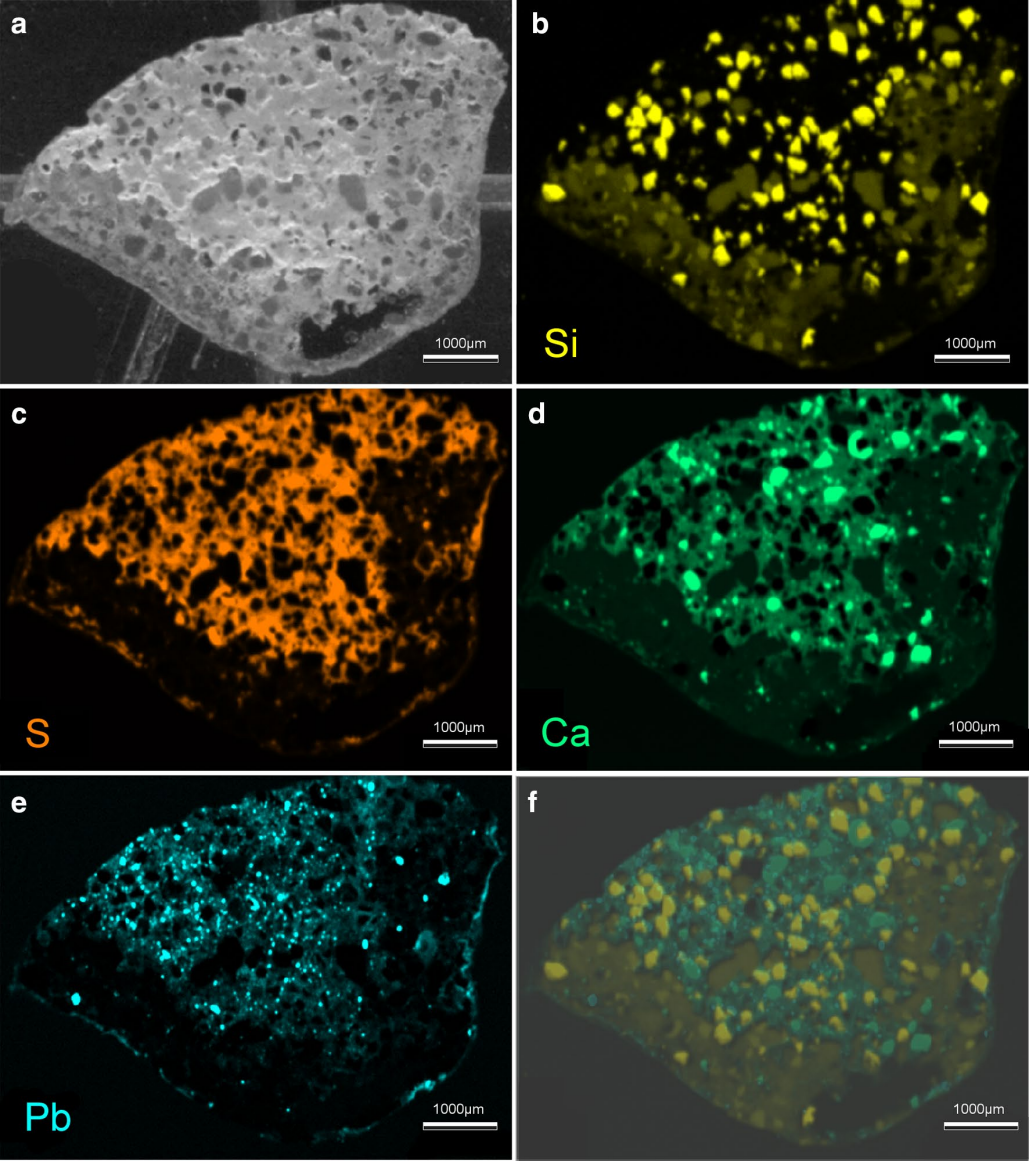
XRF Maps of M1 plaster thin section. **a** Back-scattered EM image of the sample, **b** silicon (Si) intensity map, **c** sulphur (S) intensity map, **d** calcium (Ca) intensity map, **e** lead (Pb) intensity map, **f** Combined intensity map of all four elements. Scale bars are 1000 μm

The composition of the M1 plaster was further characterised using XRF analysis, which revealed a composition much in agreement with that of the EDX analysis, with a few extra details (Fig. 7). Further sampling of M2 was deemed impossible without significantly damaging the specimen. The XRF maps show that the grains within the filler material comprise small grains (~ 0.3–0.4 mm) of sub-angular to sub-rounded quartz grains (SiO_2_) (Fig. 7b) and sub-rounded to rounded calcite grains (CaCO_3_) (Fig. 7d), these grains consisting purely of either silica or calcite. These are set within a gypsum matrix (CaSO_4_·2H_2_O), with spectra dominated by calcium and sulphur (Fig. 7c, d). Also engrained within the gypsum matrix are small grains (< 0.2 mm) containing abundant lead (Pb) (Fig. 7e). When examined under a petrological microscope and stereomicroscope, the colour and chemistry of this mineral is most consistent with the mineral minium, a reddish lead tetroxide (${\text{Pb}}^{ 2+ }_{ 2} {\text{Pb}}^{ 4+ } {\text{O}}_{ 4}$). A number of other elemental spectra were detected, including aluminium (Al), potassium (K), and manganese (Mn), but in concentrations too minor to be of significance.

### Summary

From both of these analyses, we can determine the overall general composition of the plaster filler, their relative proportions estimated from under petrological microscope. The M1 filler is composed of ~ 15–20% quartz, 30–40% calcite and the remaining fraction is the gypsum. The possible minium grains comprise < 10% of the overall fraction and are set exclusively within the gypsum plaster. The source of these minium grains is uncertain, but its presence can be explained by two theories. First, given the high density of minium (8.3 g/cm^−3^), it is possible that the compound was added to give more weight to the plaster repairs to more accurately replicate the weight of the specimen. Secondly, given minium’s historical use as a pigment, it is possible that it was added to give the plaster a more reddy-brown colour in line with the colour of the original fossil material [46]. This mineral likely represents the dense grains found within the CT scan data. A thin layer of carbon-rich coating was also applied to the surface of the plaster, likely representative of an organic shellac coating to consolidate the surface of the specimen. The origin of the quartz and calcite cannot be determined, but are not inconsistent with the composition of the Stonesfield slate from which the specimen is derived, a sandy-limestone deposit [40]. It is possible that matrix material was added to the specimen in order to both bulk the plaster and to provide closer colouration to the original fossil material, as with the addition of minium. Also notable is background chlorine contamination, either derived from environmental contamination over time or cleaning agents.

As M2 filler could not be sampled further, it must be assumed that it is of a broadly similar composition to M1 with a few notable exceptions. First is the absence of the dense grains from the CT data, strongly suggesting that minium is absent from this plaster material. Second is the presence of barium hydroxide, likely used as a sealant to prevent water permeation. Unfortunately the relative ‘stratigraphy’ and timing of the replacement plasters M1 and M2 cannot be determined, but given the significant differences in their composition, it can be assumed that the repairs were carried out at different times.

Overall, the composition is in agreement with the interpretation of the extremely preservative and conservative nature of the replacement of missing material. The presence of potential matrix material from the Stonesfield Slate and the use of minium pigment for weight and/or colour suggests that much effort was put into making the plaster match the original material as closely as possible, in much the same way as the original fragmented fossil material was set within the plaster filler to conserve as much of the original geometry of the *M. bucklandii* specimen as possible.

## Discussion

Overall, we have demonstrated that XCT in combination with chemical analysis techniques including EDX and XRF provides an excellent tool for investigating the subsurface detail of sensitive, unique and/or valuable museum specimens and can be used to assess their condition and previous conservational efforts. EDX and XRF allow conservators to assess the chemical composition of the materials, with minimal destructive sampling in EDX and typically no destructive sampling required in XRF, while XCT allows simple diagnosis of the location, volume, thickness and extent of repairs, both ensuring that appropriate measures are used for both remedial conservation and restoration efforts. It can also provide a means to re-evaluate the condition of museum objects and create a new record for objects whose conservation records have been lost, or never made, throughout its lifetime. As such, this approach could prove to be an invaluable tool for conservation approaches in museums, although there are a number of considerations that should be taken into account when using such technologies.

### Usability considerations of XCT and chemical analysis approaches

Both approaches have key advantages and drawbacks that have strong overriding influences on the kind of objects that can be imaged and what factors influence the accuracy and validity of the results. The major benefit is that they have the advantage over other internal imaging methods, such as sectioning and other forms of destructive sampling, in that they are generally non-destructive [47]. For XCT, provided the object is well-packed, the object can be examined with no direct interaction and the composition and structure of the object remains unaltered at the typically low X-ray energies used for most samples [23, 48, 49]. Chemical approaches are also typically non-invasive, macro-XRF typically utilising handheld non-contact scanners to acquire information, although micro-XRF may require samples to be taken, as in this paper, as they utilise lab-based, desktop equipment that has a limited chamber size [47, 50, 51]. EDX however will always require some minor destructive sampling, given the typically small size of the vacuum chamber utilised [47]. This leads into the relative scales at which objects can be inspected, XCT working on objects from the size of a human body, which are typically imaged in medical XCT machines, down to objects at the millimetric scale via micro-CT (μCT). [22]. The size of the object in turn dictates the resolution of the resulting volume, larger objects typically being limited to coarser resolutions (> 150 μm) while microscopic objects, particularly when imaged with synchrotron XCT, can be imaged with voxel resolutions in the nanometre range (< 1um to 10 nm), although access to these latter machines is limited and costly [22]. Macro-XRF can be used at any scale, but realistically is better for larger objects given its lower resolution compared to micro-XRF [50, 52, 53] while again, EDX is limited to samples that can be realistically attached to an SEM stub within the vacuum chamber. Additionally, XCT is potentially costly method, with purchase of a XCT machine typically being outside the price range of many institutions [54]. The ability to hire time on institutionally owned machines however means that conservators are getting better access to affordable XCT scanning solutions. XRF and EDX equipment is more accessible however, which can be found in many museum facilities and are already tools commonly utilised in other aspects of museum conservation. Another consideration is the difference between macro and micro-XRF, the latter having a wider range of elemental analysis than the former and having much higher spatial resolution, allowing more precise chemical analysis, if being more limited in the objects it can be utilised on without destructive sampling as discussed above [50, 52, 53].

For XCT, a number of further issues can also influence the quality of the final dataset. Issues during the process of scanning can cause pronounced ‘artefacts’ in the data that can disrupt interpretation and inhibit effective visualisation. These have a number of different types and causes, the most common being beam-hardening, where the outside of the scanned object has brighter grey values compared to the centre due to surface X-ray absorption [18, 55]. Others are typically caused by extremely dense components, such as metallic parts, as can be partly observed in the lead particles in Fig. 5b. These create odd shapes and structures in reconstructed data, such as streak artefacts which, if severe, can render data unusable. Issues also occur if the attenuation contrast between two materials, such as a plastic and metal, is too high. This typically results in one or the other being insufficiently penetrated, plastics being nearly completely penetrated and nearly impossible to observe in the scan data or metals having insufficient penetration [18, 55]. This means that XCT is of limited use for metallic objects as well as those with materials with highly contrasting attenuation properties. However this can be overcome using a closely related approach, neutron tomography (NT). Neutron tomography uses the same principles outlined above for scanning and reconstruction, the major difference being that is uses neutrons instead of X-rays [56, 57]. Its specific attenuation properties differ from X-rays, typically with much lower observed attenuation by metals by comparison which eliminates many of these reconstruction artefacts that occur in XCT [56, 57]. However, NT has its own drawbacks, namely in terms of its accessibility. Much like synchrotron CT, NT requires dedicated facilities to operate and there are a number throughout Europe, such as the ISIS Neutron and Muon Source (Oxfordshire) [58] and ANTARES (Bavaria) [59, 60]. As these facilities typically require application and time is limited, getting access to NT equipment is a lengthy and difficult process. Furthermore, the reliance on a nuclear reactor for neutron beam generation and the resultant size means that lab-based solutions are at this point impossible, limiting the availability of this technology which limits its potential use as a quick and useful tool in cultural heritage [59].

### Use within heritage conservation

Both chemical approaches and XCT are both, individually, common tools for analysing the conservation of many objects in cultural heritage. XCT has been used in many different contexts, including assessing the condition of sensitive objects such as scrolls [28, 54, 61, 62], furniture [17], instruments [15, 63], building materials [24, 25], sculpture [27], statuary in stone, wood and bronze [64–67], paintings [26], identifying artefacts embedded in soil blocks extracted from archaeological sites [68], identifying the contents of unlabelled plaster casts [69], identifying structures obscured by corrosion crusts [70, 71] and weaponry [72] among a substantial variety of other conservational applications within the field of cultural heritage. XRF and EDX, although XRF more so, are commonly used techniques within the field of cultural heritage and have been utilised in many applications, dominantly in assessing the condition of paintings and the materials they are made from [73, 74]. XRF has also been used in many other contexts as well however, including as stone archaeological objects [75], weaponry [76], tracing provenance of archaeological materials [77, 78], evaluating the composition of writing materials [79, 80], metallic artefacts and coins [81, 82] among a myriad of other disparate applications.

These examples demonstrate the widespread use of these methods in conservation, but much less common is their paired application. XRF is most commonly applied to paintings, which given their high aspect ratio do not normally need to be imaged using XCT [73, 74]. In cases where subsurface inspection is required, X-ray radiography is typically used [83, 84] as in [85], who utilised X-ray radiography, macro XRF and infra-red radiation (IRR) techniques to examine the subsurface structure of René Magritte’s *Le portrait*, finding another partially completed painting beneath the surface. The authors were able to characterise the composition of both artworks at the same time and better ascertain the composition of the pigments used for future conservational treatments.

Examples of the combination of these two imaging approaches are limited in number but do exist. For example, [31] demonstrate an approach that combines XCT and XRF to examine in 3D the internal and external structure and composition of natural building stones, in order to assess their weathering qualities and characterise their properties. [86] demonstrate a similar application on gypsum crusts on Lede stone, a common building material in Gothic architecture, and integrate XCT and 2D XRF among other techniques to characterise the chemical alteration of the stone through weathering. In a similar vein, [87] also integrate XCT and XRF to determine how limestone statuary behaves in response to laser cleaning, finding a difference in behaviours but no overall structural damage. [84] also demonstrate the advantages of the integration of XRF and XCT data on Baroque statuary, mapping surface XRF data onto a CT volume of wooden statue. They do however highlight some shortcomings, namely the difficulty of getting complete XRF images during scanning on obscured surfaces. A similar approach was carried out by [88] using XCT and EDX of Gothic statuary, mapping out previous restoration efforts and helping to inform key decisions on how to further conserve the object. Additionally, [89] carried out a study on surrogate papyrus phantoms to determine the usability of a number of imaging techniques in the detection of inks, utilising X-ray based methods such as XCT and XRF imaging. The authors found that both of these approaches did an excellent job of picking up dense, iron-based inks but less-dense inks were undetectable, although these carbon-based inks could be picked up using other methods, such as IRR. This suggests that even further integration of methods designed for the imaging of different materials could be of further benefit to conservation practice. Other studies that successfully integrate both of these approaches include the characterisation of pigments in furniture [90], characterisation of Roman quarried stone [91], in the characterisation of historical glass beads [92] among many other examples. The number of such studies appear to be on the rise, suggesting that the integration of XCT and chemical analysis techniques is a growing trend of high value to cultural heritage professionals.

However, the direct integration of X-ray imaging methods in a single device is also rapidly becoming a reality. The majority of these integrated systems are synchrotron facility-based and experimental, with the naturally associated issues of cost and accessibility but lab-based systems are in active development. [93] for example highlight Herakles, a cutting-edge integrated lab-based XCT, XRF tomography and XRF confocal X-ray fluorescence (cXRF) machine that allows the user to characterise the elemental composition of an object in three-dimensions, down to 1–10 μm [94]. This could overcome the need for any form of destructive sampling, but is naturally a long way from commercial accessibility at this stage. [95] also highlight an approach using cXRF which allows voxel by voxel elemental mapping of the object, a slow but time-consuming process that can be sped up by integration of initial screening from XCT data followed by focused cXRF scanning.

Overall, the combination of XCT and chemical analysis methods, such as EDX and XRF is a powerful combination that can be used in order to properly ascertain the conservational history of an object with minimal direct interaction or destructive sampling. It is being rapidly adopted and could soon become a standard tool in cultural heritage for providing complementary information about the nature of cultural heritage objects that can help to ascertain more precise information about their history and categorising undocumented conservation and restoration treatments.

## Conclusions

It has been demonstrated using the example of *M. bucklandii* Mantell, 1827 [38] that XCT combined with chemical analysis can be a potentially invaluable tool for conservation, helping museum professionals to easily identify the internal structure and composition of the artefacts in their care. This approach allows conservators and curators to readily identify and detect previous efforts to conserve objects in their care and recreate important conservational records for artefacts that may have failed to be properly recorded at the time, have been destroyed through disaster events or have gone missing over the years, although the application of this combined approach is not yet widespread but is growing in popularity.

## Abbreviations

OUMNH: Oxford University Museum of Natural History
XCT: X-ray computed tomography
Μct: micro computed tomography
EDX: energy dispersive X-ray spectroscopy
XRF: X-ray fluorescence
CT: computed tomography
NT: neutron tomography
cXRF: confocal X-ray fluorescence
IRR: infra-red radiation

## References

[1] Keene S (2002). Managing conservation in museums.

[2] Winsor P (1999). Conservation in the United Kingdom. Cult Trends.

[3] Pye E (2001). Caring for the past: issues in conservation for archaeology and museums.

[4] Edson G, Dean D (1994). The handbook for museums.

[5] Alexander EP, Alexander M (2008). Museums in motion: an introduction to the history and functions of the museum.

[6] Sully D, McCarthy C (2015). Conservation theory and practice: materials, values and people in heritage conservation. The international handbook of museum studies: museum practice.

[7] Ambrose T, Paine C (2012). Museum basics.

[8] de Guise L (2009). Practical conservation: our guide to caring for your treasures.

[9] Benton MJ, Paul GS (2000). A brief history of dinosaur paleontology. The scientific American book of dinosaurs.

[10] Fahy A, ICOM (1995). Protection, security and conservation of collections. Collections Management.

[11] Tembe G, Siddiqui S (2014). Applications of computed tomography to fossil conservation and education. Coll For.

[12] Grove L, Thomas S (2016). ‘The rhino horn on display has been replaced by a replica’: museum security in Finland and England. J Conserv Mus Stud.

[13] Fitzgerald GR (1988). Documentation guidelines for the preparation and conservation of palaeontological and geological specimens. Collect Forum.

[14] Lindsay W (1991). Mammoth Task. Cur Mus J.

[15] Sirr SA, Waddle JR (1999). Use of CT in detection of internal damage and repair and determination of authenticity in high-quality bowed stringed instruments. Radiographics.

[16] Ruffell A, Majury N, Brooks WE (2012). Geological fakes and frauds. ESR.

[17] Re A, Albertin F, Avataneo C, Brancaccio R, Corsi J, Cotto G (2014). X-ray tomography of large wooden artworks: the case study of “Doppio corpo” by Pietro Piffetti. Herit Sci.

[18] Wilson P, Williams MA, Warnett JM, Attridge A, Ketchum H, Hay J et al. Utilizing X-ray computed tomography for heritage conservation: the case of *Megalosaurus bucklandii*. I2MTC 2017 IEEE international instrumentation and measurement technology conference, Torino, Italy, 22–25 May 2017.

[19] Goldman LW (2007). Principles of CT and CT technology. J Nucl Med Technol.

[20] Kruth JP, Bartscher M, Carmignato S, Schmitt R, de Chiffre L, Weckenmann A (2011). Computed tomography for dimensional tomography. CIRP Ann Manuf Technol.

[21] Copley DC, Eberhard JW, Mohr GA (1994). Computed tomography part 1: introduction and industrial applications. J Min Met Mat Soc.

[22] Cnudde V, Boone MN (2013). High-resolution X-ray computed tomography in geosciences: a review of the current technology and applications. ESR.

[23] Warnett J, Titarenko V, Kiraci E, Attridge A, Lionheart WR (2016). Towards in-process x-ray CT for dimensional metrology. Meas Sci Technol.

[24] Cnudde V, Dubruel P, de Winne K, de Witte I, Masschaele B, Jacobs P (2009). The use of X-ray tomography in the study of water repellents and consolidants. Eng Geol.

[25] Dewanckele J, de Kock T, Fronteau G, Derluyn H, Vontobel P, Dierick M (2014). Neutron radiography and X-ray computed tomography for quantifying weathering and water uptake processed inside porous limestone used as building material. Mater Charact.

[26] Morigi MP, Casali F, Bettuzzi M, Bianconi D, Brancaccio R, Cornacchia S (2007). CT investigation of two paintings on wood table by Gentile da Fabriano. Nucl Instrum Methods Phys Res A.

[27] Badde A, Illerhaus B (2008). Three dimensional computerized microtomography in the analysis of sculpture. J Scan Microsc.

[28] Seales WB, Griffioen J, Baumann R, Field M. Analysis of Herculaneum papyri with X-ray computed tomography. 10th International conference on non-destructive investigations and microanalysis for the diagnostic and conservation of cultural and environmental heritage, Florence, Italy, 13–15 Apr 2011.

[29] Brochu CA (2000). A digitally-rendered endocast for *Tyrannosaurus rex*. J Vertebr Paleontol.

[30] Rowe T, Ketcham RA, Denison C, Colbert M, Xu X, Currie PJ (2001). The Archaeoraptor forgery. Nature.

[31] Dewanckele J, Cnudde V, Boone M, Van Loo D, De Witte Y, Pieters K (2009). Integration of X-ray microtomography and fluorescence for applications on natural building stones. J Phys Conf Ser.

[32] Johnson TRC, Krauss B, Sedlmar B, Grasruck M, Bruder H, Morhard D (2007). Material differentiation by dual energy CT: initial experience. Eur Radiol.

[33] Hidas G, Eliahou R, Duvdevani M, Coulon P, Lemaitre L, Gofrit ON (2010). Determination of renal stone composition with dual-energy CT: in vivo analysis and comparison with X-ray diffraction. Radiology.

[34] Johnson TRC, Fink C, Schönberg SO, Reiser MF (2011). Dual energy CT in clinical practice.

[35] Wagner R, Fuchs T, Scholz G, Kretzer C, Schielein R, Firsching M et al. Dual-energy computed tomography of historical musical instruments made of multiple materials. 8th conference on industrial computed tomography, Wels, Austria, 6–9 Feb 2018.

[36] McKenzie-Clark J, Magnussen J (2018). The analysis of Italian *Sigillata* potter’s stamps using dual energy computed tomography (DECT) and X-ray imaging. J Archaeol Sci Rep.

[37] Hubbell JH and Seltzer SM. Tables of X-ray mass attenuation coefficients and mass energy-absorption coefficients from 1 keV to 20 MeV for elements Z = 1 to 92 and 48 additional substances of dosimetric interest. https://www.nist.gov/pml/x-ray-mass-attenuation-coefficients. Accessed 19 Jan 2018.

[38] Mantell G (1827). Illustrations of the geology of Sussex: a general view of the geological relations of the southeastern part of England, with figures and descriptions of the fossils of Tilgate Forest.

[39] Buckland W (1824). Notice on the *Megalosaurus* or great fossil lizard of Stonesfield. Trans Geol Soc Lond.

[40] Benson RBJ (2010). A description of *Megalosaurus bucklandii* (Dinosauria: Theropoda) from the Bathonian of the UK and the relationships of Middle Jurassic theropods. Zool J Linn Soc.

[41] Benson RBJ, Barrett PM, Powell HP, Norman DB (2008). The taxonomic status of *Megalosaurus bucklandii* (Dinosauria, Theropoda) from the Middle Jurassic of Oxfordshire, UK. Palaeontology.

[42] Howlett EA, Kennedy WJ, Powell HP, Torrens HS (2017). New light on the history of *Megalosaurus*, the great lizard of Stonesfield. Arch Nat Hist.

[43] Owen R. Report on British Fossil Reptiles. Part II. Report of the eleventh meeting of the british association for the advancement of science; held at Plymouth in July 1841. London: John Murray; 1842.

[44] Owen R (1849). A history of British reptiles.

[45] Phillips J (1871). Geology of Oxford and the Valley of the Thames.

[46] Aze S, Vallet JM, Detalle V, Grauby O, Baronnet A (2008). Chromatic alterations of red lead pigments in artworks: a review. Ph Transit A Multinat J.

[47] Janssens K, Dik J, Cotte M, Susini J (2010). Photon-based techniques for nondestructive subsurface analysis of painted cultural heritage artifacts. Acc Chem Res.

[48] Abel RL, Parfitt S, Ashton N, Lewis SG, Scott B, Stringer C (2011). Digital preservation and dissemination of ancient lithic technology with modern micro-CT. Comput Gr.

[49] Abel RL, Laurinin CR, Richter M (2012). A palaeobiologist’s guide to ‘virtual’ micro-CT preparation. Palaeo Elec.

[50] Nazaroff AJ, Prujer KM, Drake BL (2010). Assessing the applicability of portable X-ray fluorescence spectrometry for obsidian provenance research in the Maya lowlands. J Arch Sci.

[51] Petrová Z, Jehlička J, Čapoun T, Hanus R, Trojek T, Goliáš V (2012). Gemstones and noble metals adorning the sceptre of the Faculty of Science of Charles University in Prague: integrated analysis by Raman and XRF handheld instruments. J Raman Spectrosc.

[52] Liritzis I, Zacharias N, Shackley MS (2011). Portable XRF of archaeological artifacts: current research, potentials and limitations. X-ray fluorescence spectrometry (XRF) in geoarchaeology.

[53] Young KE, Evans CA, Hodges KV, Bleacher JE, Graff TG (2016). A review of the handheld X-ray fluorescence spectrometer as a tool for field geologic investigations on earth and in planetary surface exploration. App Geochem.

[54] Payne EM (2012). Imaging techniques in conservation. J Conserv Mus Stud.

[55] Sun W, Brown, SB, Leach RK. An overview of industrial X-ray computed tomography. NPL Report ENG 32. Teddington: NPL: 2012.

[56] Vontobel P, Lehmann EH, Hassanein R, Frei G (2006). Neutron tomography: method and applications. Phys B.

[57] Lehmann EH, van Lang R, Estermann M, Hartmann S, LoCelso F, Kardjilov N, Kardjilov N, Festa G (2017). Bronze sculptures and lead objects tell stories about their creators: investigation of renaissance sculptures and ancient ingots by means of neutron tomography. Neutron methods for archaeology and cultural heritage.

[58] Science & Technology Facilities Council. ISIS Neutron and Muon Source. Annual Review 2017. Didcot: RTA Library; 2017.

[59] Calzada E, Schillinger B, Grünauer F (2005). Construction and assembly of the neutron radiography and tomography facility ANTARES at FRM II. Nucl Instrum Methods Phys Res A.

[60] Heinz Maier-Leibnitz Zentrum (2015). ANTARES: cold neutron radiography and tomography facility. J Larg Scale Res Facil.

[61] Mocella V, Brum E, Ferrero C, Delattre D (2015). Revealing letters in rolled Herculaneum papyri by x-ray phase-contrast imaging. Nat Commun.

[62] Bukreeva I, Mittone A, Bravin A, Festa G, Alessandrelli M, Coan P (2016). Virtual unrolling and deciphering of Herculaneum papyri by X-ray phase-contrast tomography. Nat Sci Rep.

[63] Sodini N, Dreossi D, Chen R, Fioravanti M, Giordano A, Herrestal P (2012). Non-invasive microstructural analysis of bowed stringed instruments with synchrotron radiation X-ray microtomography. J Cult Herit.

[64] Morigi MP, Casali F, Bettuzzi M, Brancaccio R, D’Errico V (2010). Application of X-ray computed tomography to cultural heritage diagnostics. Appl Phys A.

[65] Pavel C, Suciu C, Constantin F, Bugoi R (2013). X-ray computed tomography investigations of Cucuteni ceramic statuettes. Doc Prae XL.

[66] Osterloh KRS, Nusser A. X-ray and neutron radiological methods to support the conservation of wooden artworks soaked with a polluting impregnant “Carbolineum”. 11th European Conference on Non-Destructive Testing (ECNDT 2014), Prague, Czech Republic, 6–10 Oct 2014.

[67] Bettuzzi M, Casali F, Morigi MP, Brancaccio R, Carson D, Chiari G (2015). Computed tomography of a medium size Roman bronze statue of Cupid. Appl Phys A.

[68] Stelzner J, Ebinger-Rist N, Peek C, Schillinger B (2010). The application of 3D computed tomography with X-rays and neutrons to visualize archaeological objects in blocks of soil. Stud Conserv.

[69] Schilling R, Jastram B, Wings O, Schwarz-Wings D, Issever AS (2013). Reviving the dinosaur: virtual reconstruction and three-dimensional printing of a dinosaur vertebra. Radiology.

[70] Haneca K, Deforce K, Boone MN, van Loo D, Dierick M, van Acker J (2012). X-ray sub-micron tomography as a tool for the study of archaeological wood preserved through the corrosion of metal objects. Archaeometry.

[71] Mearns DL, Parham D, Frohlich B (2016). A Portuguese East Indiaman from the 1502–1503 Fleet of Vasco da Gama off Al Hallaniyah Island, Oman: an interim report. Int J Naut Archaeol.

[72] Mannes D, Schmid F, Frey J, Schmidt-Ott K, Lehmann E (2014). Combined neutron and X-ray imaging for non-invasive investigations of cultural heritage objects. Phys Proc.

[73] Dik J, Janssens K, Van Der Snickt G, Van Der Loeff L, Rickers K, Cotte M (2008). Visualization of a lost painting by Vincent van Gogh using synchrotron radiation based X-ray fluorescence elemental mapping. Anal Chem.

[74] Walter P, Sarrazin P, Gailhanou M, Hérouard D, Verney A, Blake D (2018). Full-field XRF instrument for cultural heritage: application to the study of a Caillebotte painting. Xray Spectrom.

[75] Cueva AM, Bernardini F, Gianoncelli A, Tuniz C (2015). Energy dispersive X-ray diffraction and fluorescence portable system for cultural heritage applications. Xray Spectrom.

[76] Gharib A, Mohamed H, Abdel Ghany N (2018). Nondestructive techniques in the study of a gilded metallic sword from the Islamic Art Museum. Egypt J Archaeol Restor Stud.

[77] Germinario L, Zara A, Maritan L, Bonetto J, Hanchar JM, Sassi R (2017). Tracking trachyte on the Roman routes: provenance study of Roman infrastructure and insights into ancient trades in northern Italy. Geoarchael Int J.

[78] Kasztovszky Z, Maróti B, Harsányi I, Párkányi D, Szilágyi V (2018). A comparative study of PGAA and portable XRF used for non-destructive provenancing archaeological obsidian. Quat Int.

[79] Cohen Z, Olszowy-Schlanger J, Hahn O, Rabin I, Wandrey I (2017). Composition analysis of writing materials in Geniza fragments. Jewish manuscript cultures: perspectives from palaeography, codicology, provenance history, material analysis and art history.

[80] Duh J, Krstić D, Desnica V, Fazinić S (2018). Non-destructive study of iron gall inks in manuscripts. Nucl Instrum Methods Phys Res B.

[81] Fierascu I, Fierascu RC, Ortan A, Mirea DA, Morarescu C (2017). Integrated methodology for the non-destructive characterization of cultural heritage artifacts. Rom Rep Phys.

[82] Herringer SN, Ryzewski K, Bilheux HZ, Bilheux JC, Sheldon BW (2018). Evaluation of segregation in Roman sestertius coins. J Mater Sci.

[83] Zemlicka J, Jakubek J, Kroupa M, Hradil D, Hradilova J, Mislerova H. Analysis of painted arts by energy sensitive radiographic techniques with the Pixel Detector Timepix. 12th International workshop on radiation imaging detectors 2011, Cambridge, UK, 11–15 July 2010.

[84] Vavřik D, Kumpová I, Vopálensky M, Lautenkranc J. Analysis of baroque sculpture based on X-ray fluorescence imaging and X-ray computed tomography data fusion. 7th Conference on industrial computed tomography, Leuven, Belgium 7–9 Feb 2017.

[85] Van der Snickt G, Martins A, Delaney J, Janssens K, Ziebel J, Duffy M (2016). Exploring a Hidden painting Below the Surface of René Magritte’s *Le Portrait*. Appl Spectrosc.

[86] de Kock T, Dewanckele J, Boone M, Vincze L, Fronteau G, Van Hoorebeke L. Multidisciplinary characterization of gypsum crust on Lede stone (Belgium). 12th International congress on the deterioration and conservation of stone. Columbia University, New York. 21–25 Oct 2012.

[87] Senesi GS, Allegretta I, Porfido C, De Pascale O, Terzano R (2017). Application of micro X-ray fluorescence and micro computed tomography to the study of laser cleaning efficiency on limestone monuments covered by black crusts. Talanta.

[88] Mikolajska A, Walczak M, Kaszowska Z, Zawadzka MU, Banyś RP (2012). X-ray techniques in the investigation of a Gothic sculpture: the risen christ. Nukleonika.

[89] Gibson A, Piquette KE, Bergman U, Christens-Barry W, Davis G, Endrizzi M (2018). An assessment of multimodal imaging of subsurface text in mummy cartonnage using surrogate papyrus phantoms. Herit Sci.

[90] Burgio L, Melchar D, Strekopytov S, Peggie DA, Di Crescenzo MM, Keneghan B (2018). Identification, characterisation and mapping of calomel as ‘mercury white’, a previously undocumented pigment from South America, and its use on a *barniz de Pasto* cabinet at the Victoria and Albert Museum. Micro J.

[91] Lanzón M, Cnudde V, De Kock T, Dewanckele J, Piñero A (2014). X-ray tomography and chemical-physical study of the calcarenite extracted from a Roman quarry in Catagena (Spain). Eng Geol.

[92] Ngan-Tillard DJM, Huisman DJ, Corbella F, Van Nass A (2018). Over the rainbow? Micro-CT scanning to non-destructively study Roman and early medieval glass bead manufacture. J Archaeol Sci.

[93] Laforce B, Massschaele B, Boone MN, Schaubroeck D, Dierick M, Vekemans B (2017). Integrated Three-dimensional microanalysis combining X-ray microtomography and X-ray fluorescence methodologies. Anal Chem.

[94] Boone MN, Laforce B, Masschaele B, Dierick M, Van Assche F, Cnudde V (2018). Combined chemical and morphological characterization using innovative lab-based equipment. Microsc Microanal.

[95] Cordes NL, Seshadri S, Havrilla GJ, Yuan X, Feser M, Patterson BM (2014). Three dimensional subsurface identification of minerals using confocal micro X-ray fluorescence and Micro X-ray computed tomography. Spectrochim Acta Part B At Spectrosc.

